# SNO pediatric molecular tumor board series: Successful beginnings and future directions

**DOI:** 10.1093/noajnl/vdae076

**Published:** 2024-05-21

**Authors:** Holly B Lindsay, Craig Erker, Suzanne Laughlin, Annie Huang

**Affiliations:** Children’s Hospital Colorado Center for Cancer and Blood Disorders, University of Colorado Anschutz Medical Campus School of Medicine, Aurora, Colorado, USA; Deparment of Pediatrics, Division of Pediatric Hematology/Oncology, Dalhousie University and IWK Health Centre, Halifax, Nova Scotia, Canada; Department of Medical Imaging, University of Toronto, Toronto, Ontario, Canada; Department of Diagnostic and Interventional Radiology, The Hospital for Sick Children, Toronto, Ontario, Canada; Department of Laboratory Medicine and Pathobiology, Faculty of Medicine, University of Toronto, Toronto, Ontario, Canada; Division of Haematology/Oncology, Department of Pediatrics, Hospital for Sick Children, Toronto, Ontario, Canada; Arthur and Sonia Labatt Brain Tumor Research Centre, Hospital for Sick Children, Toronto, Ontario, Canada

An ongoing objective of the Society for Neuro-Oncology (SNO) is to expand the role and engagement of pediatrics in the society and to provide an academic home for physicians in the field of pediatric neuro-oncology. With the support of SNO leadership and the 3 chairs of the newly formed SNO Pediatrics Special Interest Track, we established the SNO Pediatric Molecular Tumor Board Series in 2021. The mission of the tumor board is to educate attendees on the clinical management of pediatric brain and spinal cord tumors in the context of World Health Organization molecularly based diagnostics and to foster worldwide clinical and research collaborations within pediatric neuro-oncology. We have presented 8 quarterly pediatric sessions in this series, with each tumor board focusing on a specific tumor diagnosis; topics covered thus far have been atypical teratoid rhabdoid tumor, craniopharyngioma, embryonal tumor with multilayered rosettes, hypermutant CNS tumors, infant medulloblastoma, *NTRK*-altered CNS tumors, pineoblastoma, and posterior fossa ependymoma. Each presentation is recorded and uploaded to SNO’s Neuro-Oncology Academy (https://neuro-onc-academy.org/) where they are available for free access for both SNO members and nonmembers.

A terms-of-reference document has been established for these sessions. Each tumor board totals 90 minutes and is presented virtually following a defined outline. Using a standard single-slide template focusing on patient presentation, treatment course, and patient outcome, 3–4 cases of patients <25 years old at diagnosis are presented by a member of the primary pediatric neuro-oncology team in a deidentified manner. The tumor board is educational rather than a working meeting; consequently, each case must have at least 6 months of postdiagnosis follow-up to be considered for presentation. Additionally, as part of our commitment to equity and global inclusivity, it is a goal to ensure that case presentations from low- or middle-income countries and countries outside of North America and Europe are highlighted. After each case presentation, the relevant anonymized imaging from each patient is shown and discussed by an expert pediatric neuroradiologist followed by tumor pathology and molecular review by a leading pediatric neuropathologist. All presented cases should have molecular testing. One of the tumor board leaders next presents focused education on the tumor including incidence, biology, treatment options, outcomes, and active research. The second half of the tumor board is a question-and-answer session. Questions are posed by attendees of the virtual meeting and responded to verbally by a member of the expert panel chosen by the tumor board organizers. The expert panel is typically comprised of 2–4 pediatric neuro-oncologists, a pediatric neurosurgeon, and a pediatric radiation oncologist.

**Figure 1.**
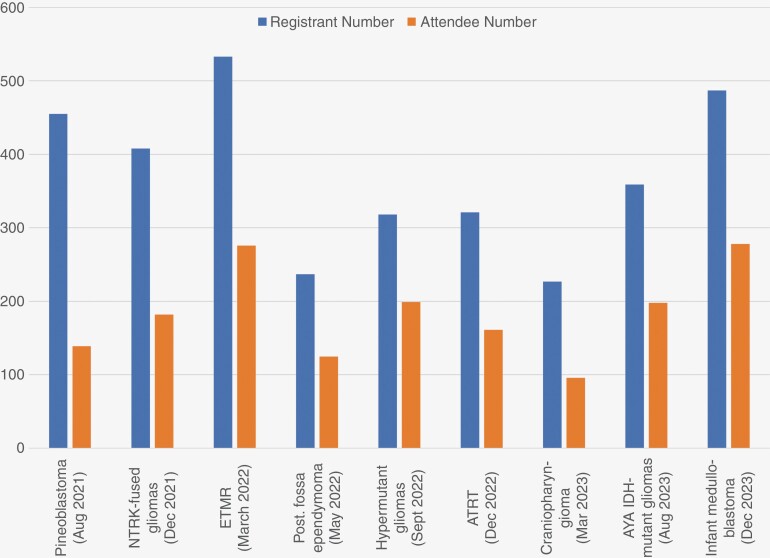
SNO pediatric molecular tumor board engagement.

Over the past 2 years, the Pediatric Molecular Tumor Board has become SNO’s most popular series and has generated significant interest and engagement within the pediatric neuro-oncology community. The series has had an average of over 340 registrants for each live broadcast (Figure 1), importantly including significant international representation. To improve interactions and collaborations with adult neuro-oncologists and based upon high levels of international engagement, neuro-oncologists focused on adolescent and young adult tumors (AYA) tumors recently led the first AYA-focused tumor board on *IDH*-mutant gliomas completed in August 2023. AYA cases are planned to be presented at least semiannually as part of this series. In addition, the SNO administration team plans to provide continuing medical education credits for attendees of the series. Overall, the quality and consistent attendance of the SNO Pediatric Molecular Tumor Board highlights the importance of pediatric-focused clinicians and researchers to the society.

